# Proton Pump Inhibitor-Induced Hypomagnesemia: A Rare, Potentially Fatal Complication

**DOI:** 10.7759/cureus.8191

**Published:** 2020-05-18

**Authors:** Alec J Pawlukiewicz, Matthew Geringer, David Dado, Daniel Nassery

**Affiliations:** 1 Emergency Medicine, Brooke Army Medical Center, Fort Sam Houston, USA; 2 Internal Medicine, Brooke Army Medical Center, Fort Sam Houston, USA; 3 Nephrology, Brooke Army Medical Center, Fort Sam Houston, San Antonio, USA

**Keywords:** tremor, proton pump inhibitor, hypomagnesemia

## Abstract

Hypomagnesemia has been associated with a variety of abnormalities, including neurological, cardiac and secondary electrolyte abnormalities. We present the case of a 77-year-old male who presented to the emergency department with tremor and difficulty walking and was found to have severe hypomagnesemia necessitating hospital admission. After thorough workup, the patient's hospital course concluded that the profound hypomagnesemia was secondary to proton pump inhibitor use. Physicians should be aware of proton pump inhibitor-induced hypomagnesemia as a rare, but easily correctable etiology of hypomagnesemia.

## Introduction

Hypomagnesemia, a condition characterized by low serum magnesium levels, can result in fatal complications, including tetany, seizures, cardiac arrhythmias and secondary electrolyte abnormalities [1]. The seriousness of the possible complications of hypomagnesemia highlights the necessity of proper magnesium repletion and identification of the underlying etiology. We present a case of severe hypomagnesemia presenting with generalized tremor found to be secondary to chronic proton pump inhibitor (PPI) therapy.

## Case presentation

**Figure 1:**
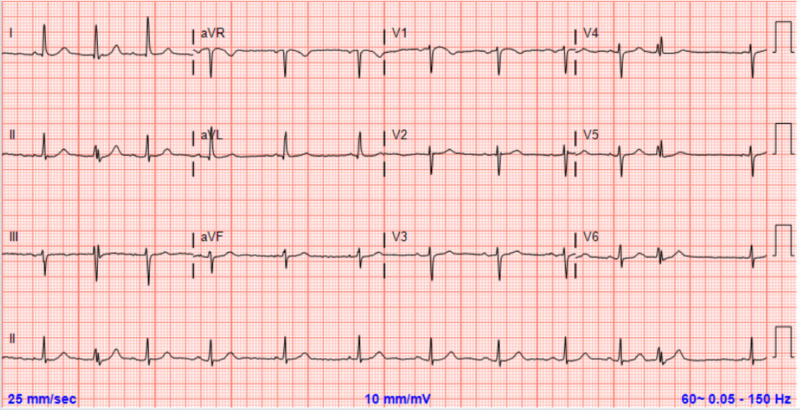
Patient’s electrocardiogram demonstrating multiple premature atrial contractions.

A 77-year-old gentleman with a past medical history significant for hypertension, hyperlipidemia, chronic kidney disease and recent cerebrovascular accident presented to the emergency department with two weeks of intention tremors increasing in intensity to the extent that he was unable to walk. He also reported limited oral intake secondary to nausea over approximately the same period. Medication reconciliation was notable for omeprazole and furosemide. Vital signs in the emergency department were within normal limits, and physical exam was notable only for significant generalized tetany that worsened with intention. The patient’s electrocardiogram (EKG) revealed normal sinus rhythm with multiple premature atrial contractions (Figure 1).

Initial laboratory results showed a creatinine of 1.18 mg/dL (which is at the patient’s baseline), sodium of 143 mmol/L, potassium of 3.3 mmol/L, phosphate of 2.5 mg/dL, calcium of 7.4 mg/dL and an undetectable magnesium level of <0.6 mg/dL. Additionally, parathyroid hormone was elevated to 79 pg/mL (reference range = 15-65 pg/mL).

**Figure 2:**
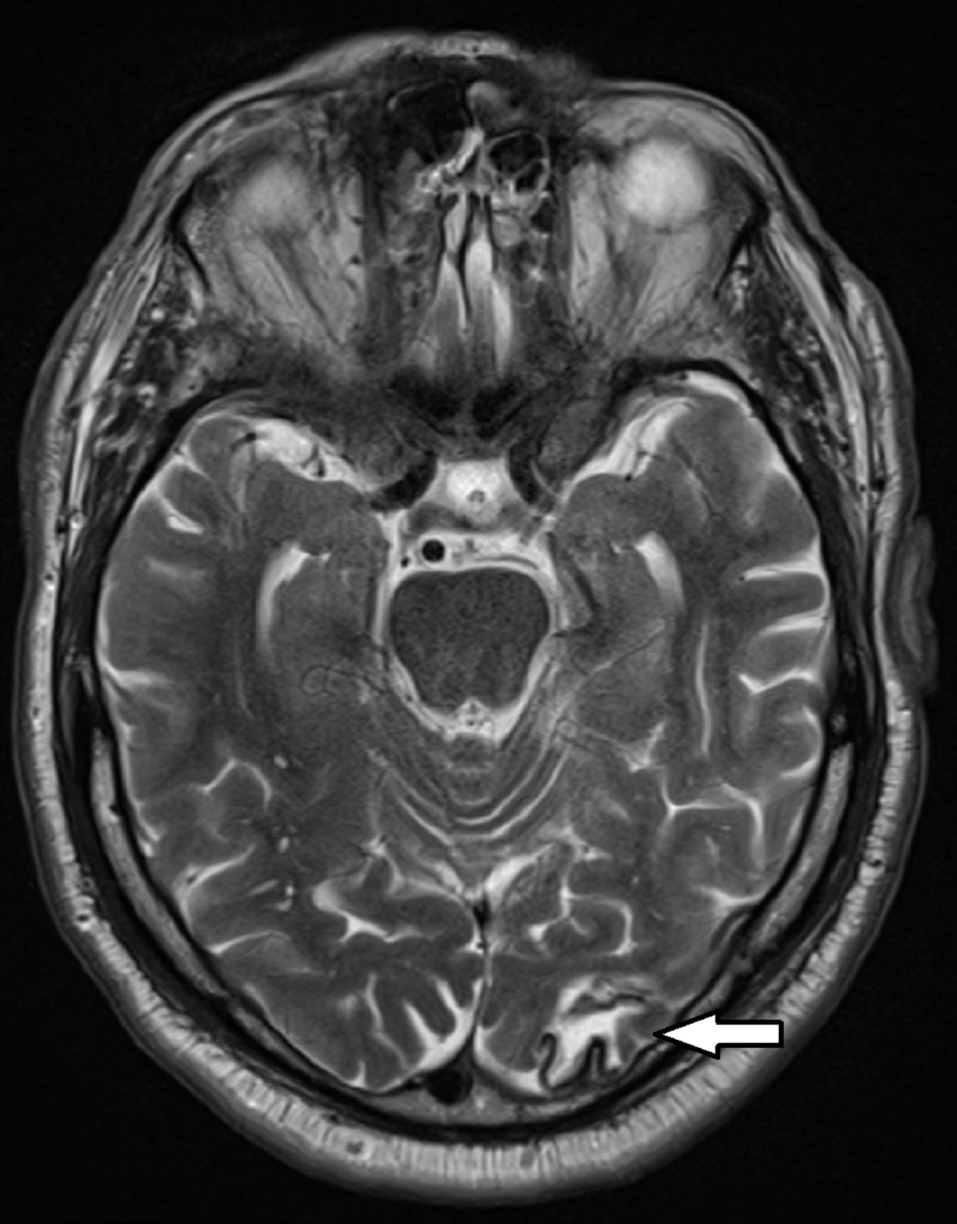
Patient’s magnetic resonance imaging showing no acute infarct and encephalomalacia of the left occipital lobe (arrow), consistent with the chronic appearance of prior infarct.

In the emergency department, he was given 20 milliequivalents oral potassium replacement and one gram of intravenous magnesium sulfate with immediate improvement of his tetany. Omeprazole and furosemide were discontinued on admission, and causes of hypomagnesemia were evaluated. Both random urine and 24-hour urine magnesium testing did not reveal evidence of renal magnesium wasting. Furthermore, in the setting of recent occipital and cerebellar infarcts, magnetic resonance imaging of the patient’s brain was performed, which showed only encephalomalacia of the left occipital region from his prior ischemic stroke without evidence of an acute infarct as possible etiology (Figure 2).

The patient required aggressive electrolyte replacement of magnesium, calcium, phosphate and potassium throughout his hospital course. He was asymptomatic at the time of discharge on hospital day 5 and was prescribed oral magnesium, calcium and vitamin D replacement. Discharge laboratory results were notable for stable creatinine, sodium of 139 mmol/L, potassium of 4.3 mmol/L, phosphate of 2.4 mg/dL, calcium of 8.4 mg/dL and a magnesium level of 1.8 mg/dL. Repeat magnesium level one week after discharge remained stable at 2.2 mg/dL. The patient remained on magnesium supplemental for a period of eight months after discharge before this was discontinued by his primary care physician. His measured serum magnesium level three months after discontinuation was within normal limits at 2.1 mg/dL.

## Discussion

The clinical effects of hypomagnesemia can be classified into the three general categories, including neurological manifestations, cardiovascular effects and secondary electrolyte abnormalities. The neurological manifestations of hypomagnesemia reflect a state of neuroexcitability and include abnormal movements, spasms, tetany, tremor, seizures and coma [1]. The neurological effects are believed to be the result of the effect of magnesium on the glutamate receptor. Magnesium acts as a competitive inhibitor of calcium at the neuromuscular junction [2]. Decreased magnesium levels result in an increased calcium influx leading to an increase in glutamate release and resultant neuroexcitability [3]. Hypomagnesemia has also been shown to result in a decreased threshold for axonal depolarization, which may also contribute to the state of neuroexcitability [4].

The major cardiovascular effect of hypomagnesemia levels is a variety of arrhythmias, including premature atrial contractions, supraventricular tachycardia, atrial fibrillation and ventricular tachydysrhythmias [1]. Further, EKG changes typically associated with hypomagnesemia include PR and QT segment prolongation [1]. The primary mechanism for the arrhythmogenicity of hypomagnesemia is via decreased activity of sodium-potassium-adenosine triphosphatase (Na/K ATPase) [5]. Because magnesium is a required cofactor for the Na/K ATPase, hypomagnesemia leads to a decrease in its activity level and subsequent increase in myocardial irritability.

The two primary electrolyte abnormalities associated with hypomagnesemia include hypokalemia and hypocalcemia. Hypokalemia in the setting of hypomagnesemia typically occurs due to decreased activity of the renal outer medullary potassium channel (ROMK) in the distal convoluted tubule and cortical collecting duct. Magnesium acts as an inhibitor of the ROMK, which is responsible for secretion of potassium; thus, decreased magnesium levels result in an increase in potassium excretion [6,7]. This may also be exacerbated by a concomitant increase in distal sodium delivery or increase in aldosterone levels [6]. The mechanism by which hypomagnesemia causes hypocalcemia is typically through both a decrease in parathyroid hormone (PTH) secretion from the parathyroid gland and an increased resistance of the tissues to PTH [8,9]. The elevated PTH seen in our case was likely secondary to the increased tissue resistance cause by hypomagnesemia.

The body’s total stores of magnesium are located primarily within bone and muscle cells with only about 1% circulating in the blood [10-12]. Magnesium absorption takes place primarily in the small and large bowels through both active and passive transport [12]. The passive transport of magnesium takes place via the transfusion of magnesium between enterocytes in a concentration-dependent manner [12-14]. Active magnesium transport occurs primarily in the cecum and large intestine via transient receptor potential melastin 6 and 7 (TRPM6/7) [12-14]. Due to the high affinity of these ion channels, active transport is of particular importance to the maintenance of magnesium stores during times of decreased dietary magnesium, and subsequent decreased passive absorption [14].

Renal reabsorption of magnesium occurs primarily in the distal convoluted tubule via passive transportation [15]. Additionally, active transportation takes place in the distal convoluted tubule via TRPM6/7 [13,14]. Hypomagnesemia results in an increase in the expression of TRPM6/7, resulting in significantly increased active reabsorption by the kidneys [13,16].

Because there is no hormonal axis directly related to magnesium homeostasis, hypomagnesemia tends to result from alterations to the absorption and secretion of magnesium via the gastrointestinal and renal systems. Due to the large intracellular stores of magnesium relative to the amount circulating in the blood, considerable depletion of the intracellular stores may occur prior to the occurrence of decreased serum concentrations and the resulting symptoms [17].

The association between PPIs and hypomagnesemia was first reported in 2006 in a case presenting with carpopedal and truncal spasm in two patients on omeprazole [18]. Subsequently, cases of PPI-induced hypomagnesemia involving other PPIs have been reported, indicating a class effect [19]. The proposed mechanism for PPI-induced hypomagnesemia involves TRPM6/7 [14]. PPIs are believed to decrease the binding affinity of magnesium for TRMP6/7 via alterations to the pH of the intestinal lumen, thereby impairing the active transportation of magnesium in the gastrointestinal tract [20]. PPIs have not been shown to result in any alterations to passive gastrointestinal absorption, which occurs via a mechanism based on simple diffusion and is not influenced by luminal pH [16]. Furthermore, in cases of PPI-induced hypomagnesemia, renal excretion of magnesium is typically appropriately reduced [16]. Reports of PPI-induced hypomagnesemia typically occur in patients who have taken PPIs over the course of a prolonged period of time [14]. This is likely secondary to a slight net negative balance between the absorption and secretion of magnesium slowly depleting the intracellular magnesium stores.

Our case is consistent with these prior case reports and the proposed mechanism for PPI-induced hypomagnesemia. The patient described in our case had been taking omeprazole for several years and presented with neurological, cardiac and secondary electrolyte manifestations of hypomagnesemia. He demonstrated appropriately reduced urinary magnesium excretion consistent with a gastrointestinal etiology of hypomagnesemia and maintained stable magnesium levels after his PPI was discontinued.

## Conclusions

PPIs may result in a clinically significant decrease in serum magnesium levels with potentially harmful and even deadly effects. Prescribing physicians should be aware of this possible adverse effect and maintain a high clinical suspicion for hypomagnesemia in patients on PPIs. Furthermore, consideration should be given to the screening of serum magnesium levels in patients taking PPIs, especially those with concomitant risk factors for developing hypomagnesemia.
